# Supplementary figures and images for: Sedentary Behavior Impacts on the Epigenome and Transcriptome: Lessons from Muscle Inactivation in Drosophila Larvae
Source: Cells. 2023 Sep 22;12(19):2333. doi: 10.3390/cells12192333 (PMC10571804; doi:10.3390/cells12192333)

A

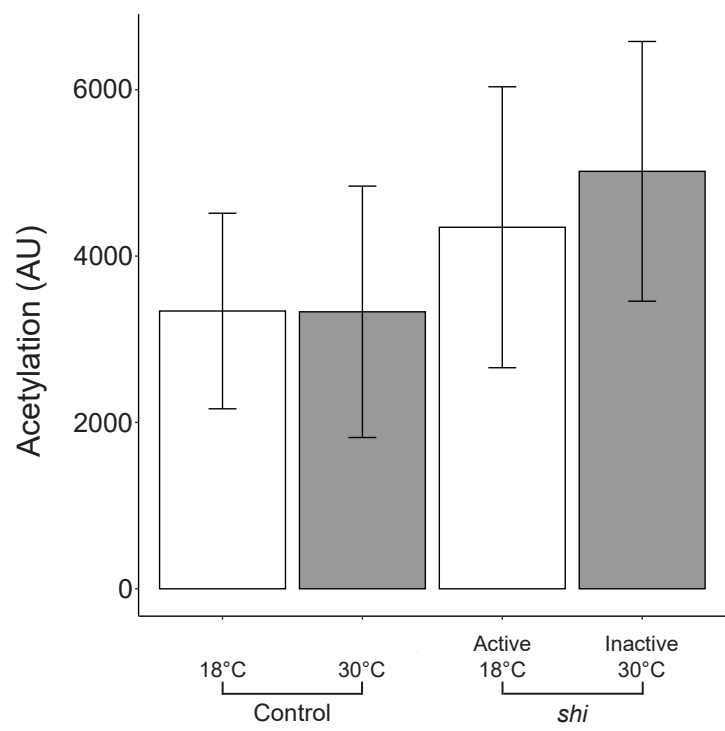

B

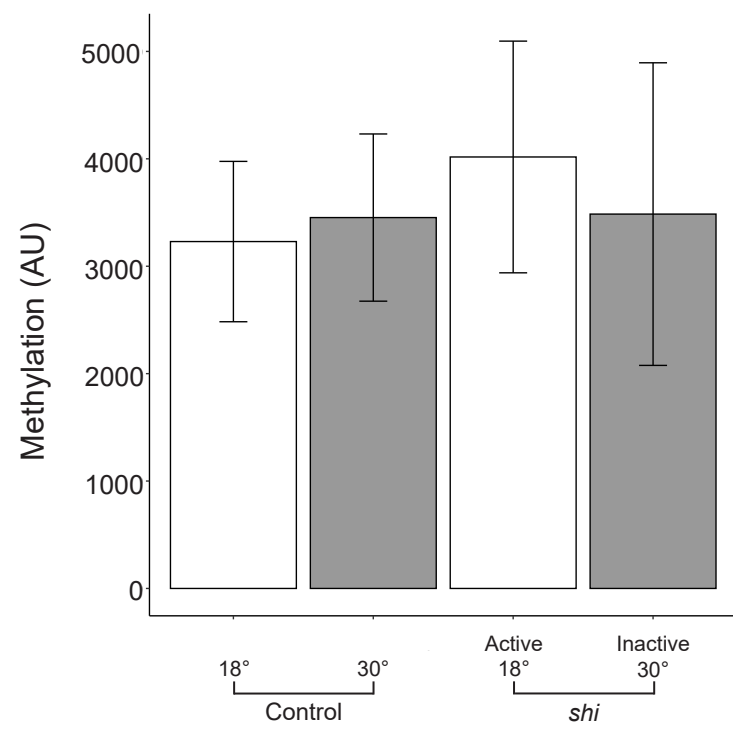

Supplement: Supplementary file 1 [file cells-12-02333-s001.zip › Supplement information/Supp Fig 1/Fig 1 supp.pdf]
